# The Potential Role of Dietary Polyphenols in the Prevention and Treatment of Acute Leukemia

**DOI:** 10.3390/nu16234100

**Published:** 2024-11-28

**Authors:** Fatemeh Fakhar, Kiana Mohammadian, Shayan Keramat, Agata Stanek

**Affiliations:** 1 Division of Hematology and Blood Banking, Department of Medical Laboratory Sciences, School of Paramedical Sciences, Shiraz University of Medical Sciences, Shiraz 71348, Iran; fatemehfakhar1999@gmail.com (F.F.); kiana.mohammadian78@gmail.com (K.M.); 2 VAS-European Independent Foundation in Angiology/Vascular Medicine, Via GB Grassi 74, 20157 Milan, Italy; shayan.sk1993@gmail.com; 3 Support Association of Patients of Buerger’s Disease, Buerger’s Disease NGO, Mashhad 9183785195, Iran; 4 Department of Internal Medicine, Metabolic Diseases and Angiology, Faculty of Health Sciences in Katowice, Medical University of Silesia, Upper Silesian Medical Center, Ziołowa 45/47 St., 40-055 Katowice, Poland

**Keywords:** dietary polyphenols, acute leukemia, anticancer, anti-inflammatory, antioxidant

## Abstract

Acute leukemia is a prevalent cancer worldwide and is classified into two distinct forms. Currently, various therapies have been developed for this disease; however, the issues of recurrence, resistance to treatment, and adverse effects require the exploration of novel treatments. Polyphenols, classified into four categories, are secondary metabolites originating from plants that demonstrate diverse metabolic features such as anticancer, anti-inflammatory, and antioxidant activities. Consequently, they have attracted attention for therapeutic and preventive measures. Research indicates that dietary polyphenols can mitigate the disease burden of acute leukemias by influencing the molecular pathways associated with the disease and its inflammatory processes. Furthermore, owing to their antioxidant properties, they can reduce the amounts of reactive oxygen species generated from the disrupted molecular pathways in these malignancies. The therapeutic actions of polyphenols can facilitate disease recovery by interrupting the cell cycle and causing apoptosis by activating pro-apoptotic genes. In conclusion, the intake of dietary polyphenols, due to their convenience and availability, coupled with the positive outcomes associated with their use in conjunction with conventional therapies, may function as an efficient therapeutic and preventive measure for acute leukemia.

## 1. Introduction

Leukemia is among the most prevalent malignancies affecting humans globally [1]. The worldwide incidence of leukemia is 474,519 cases, including 67,784 cases in North America. The age-standardized incidence rate is approximately 11 per 100,000, while the death rate is approximately 3.2 per 100,000 [2]. Acute leukemia is a malignant clonal disorder characterized by abnormal immature and undifferentiated hematopoietic cells in the bone marrow. A lymphoid, myeloid, mixed, or undifferentiated hematopoietic cell origin is the criterion for disease classification in these diseases [1]. Although there have been many substantial advances in the management of acute leukemia, the pathogenesis of these illnesses still remains unidentified. Undoubtedly, inheritance significantly influences the progression of these disorders [1]. The American Cancer Society’s Cancer Facts and Figures predicts that out of the 1,806,590 new cancer cases in the US each year, 178,520 will be diagnosed with leukemia, lymphoma, or myeloma, accounting for 9.9 percent of the total [3].

Acute myeloid leukemia (AML) is a diverse blood cancer characterized by the defective development of bone marrow myeloid progenitors. AML is a clonal process arising from a modified hematopoietic stem cell (HSC), leading to clonal proliferation in otherwise healthy individuals, which precedes the onset of leukemia [4]. Acute lymphoblastic leukemia (ALL) is the neoplastic growth of lymphoid progenitors which invade the bone marrow and have the potential to spread to areas outside of the bone marrow. The B cell lineage constitutes 85% of this malignancy, the T cell lineage accounts for 10 to 15%, and the natural killer (NK) cell lineage represents less than 1% of rare instances [5]. Among children and adolescents, it is the most common malignancy, comprising approximately 25–30% of all juvenile malignancies [1,2].

Currently, the usual treatment for acute leukemias encompasses conventional chemotherapy, targeted therapy, hematopoietic stem cell transplantation (HSCT), and immunotherapy. However, these therapies frequently result in adverse effects, resistance to treatment, and recurrence [6,7,8]. For example, the administration of corticosteroids in the management of ALL can induce neurophysiological consequences, while chemotherapy can lead to complications including immunosuppression and cardiac abnormalities [9]. This underscores the need for novel therapeutic strategies. It has been indicated that dietary polyphenols may serve as therapeutic agents in leukemia treatment in acute leukemias, while inflammation and oxidative stress significantly influence their onset, development, and resistance to treatment by affecting the tumor microenvironment [10,11]. Because of their antioxidant and anti-inflammatory properties, polyphenols may be able to alleviate these concerns [12,13]. They show anticancer capabilities via regulating carcinogenesis pathways, including inducing cell cycle arrest and apoptosis, reducing tumor development, and enhancing therapeutic outcomes when used in conjunction with existing treatments [12,14].

Polyphenols, secondary metabolites derived from plants, are prominently found in the daily diet. Their consumption, in addition to exhibiting curative and protective capabilities, has demonstrated therapeutic advantages, including a reduction in the risk of diabetes and neurological disorders [15]. Due to their accessibility, minimal toxicity, and user-friendliness, they are considered a viable therapy alternative [16]. Phenolic chemicals are commonly found in foods including fruits, vegetables, nuts, seeds, cereals, honey, royal jelly, cocoa goods, and olive oil, and they are also found in beverages including coffee, tea, beer, and wine [15,17,18]. There are four primary types of polyphenols, classified by the quantity of phenol rings and the integrating elements: flavonoids, phenolic acids, lignans, and stilbenes [19]. They exist in both soluble and insoluble forms and are organic molecules characterized by hydroxyl groups and aromatic rings, which determine their structure and dictate their properties [20].

There are abundant data that elucidate the health advantages of dietary polyphenols, including their antioxidant, anticancer, and anti-inflammatory characteristics. In this research, the numerous health advantages of polyphenols found in food, which aid in the management and avoidance of acute leukemia, are our primary area of interest.

## 2. Quantitative Content of Dietary Polyphenols

Dietary polyphenols consist of several phenolic substances. Blueberries comprise 6.6% phenolic acids, 12.9% flavonoids, and 2.7% anthocyanidins [21]. Green tea typically comprises 169–273 mg GAE/g (gallic acid equivalent) of polyphenols, including gallocatechin gallate, gallocatechins, epigallocatechin, epicatechin-3-gallate, epicatechin, and epigallocatechin-3-gallate (EGCG) [22,23]. In contrast, black tea comprises 262 mg of polyphenols per cup, with 65 mg attributed to particular polyphenols [24]. Consequently, green tea contains a higher concentration of polyphenols and may exert more significant effects than black tea [25]. The polyphenolic composition of apples includes anthocyanin (0.1–6.5 mg), chlorogenic acid (10.6–80.3 mg), flavan-3-ols (19.6 and 55.8 mg), phloridzin (1.0–9.3 mg), and flavonols (17.7–33.1 mg) [26]. The various components of grapes have differing quantities of polyphenols. The skin and leaf possess 374.6 and 351.6 mg/g GAE (gallic acid equivalent) of polyphenols, whilst the flesh and seeds contain 23.8 and 2178.8 mg/g GAE, respectively [27]. In terms of the polyphenol concentration, a standard wine bottle typically contains between 0.2 and 0.3 g/L [28].

The impact of polyphenols is contingent upon the quantity ingested and the degree of absorption. Grape seeds possess a greater concentration of polyphenols than other chemicals, with green tea ranking next, potentially exhibiting superior efficacy compared to other substances. The polyphenolic composition of apples has greater variability than their total polyphenol content, suggesting that they are a valuable source of polyphenols. Additionally, blueberries can serve as a significant source of polyphenols owing to their varied composition. Wine contains a lower concentration of polyphenols compared to other chemicals and may exhibit lesser effects [29].

## 3. Antioxidant Properties of Polyphenols

Oxidative stress is regarded as a significant element in the biology of aging and diseases, such as cardiovascular disease, type II diabetes, and cancer [30,31]. Reactive oxygen species (ROS)—the most frequent type of free radical—are primarily responsible for causing damage to biological components such proteins, lipids, and DNA [30,32]. To manage the surplus of free radicals generated during oxidative stress reactions, humans have evolved both endogenous and external strategies to maintain the redox equilibrium [30]. Dietary polyphenols are currently being extensively studied for their potent antioxidant capacities and their regulatory effects on cellular activity [30]. Variations in polyphenol types arise from differences in their nucleus structures, substituent types, and substituent positions, leading to disparities in antioxidant efficacy [33]. To counteract and mitigate the harmful effects of ROS, various antioxidant techniques have been developed, either by increasing organic antioxidant enzyme defense or by strengthening nonenzymatic defenses [34]. Dietary polyphenols are documented to exhibit significant antioxidant activity through endogenous and exogenous processes [30]. These powerful antioxidant polyphenols can donate an electron or a hydrogen atom to free radicals, making them harmless [35]. The phenolic hydroxyl group is crucial for the capacity to neutralize free radicals, with the quantity and positioning of hydroxyl and other groups being significant influencing factors [33]. In order to slow down the oxidation rate, polyphenols limit the creation of free radicals by either blocking their formation or neutralizing dangerous species and free radical precursors. In the process of lipid peroxidation reactions, they also act as chain breakers or direct radical scavengers. Free radicals can be neutralized and transformed into more stable free radicals by adding an electron to them; this process is called a chain break [36,37]. Polyphenols exhibit antioxidant action not just through their radical scavenging characteristics but also via iron binding. As with other transition metals, iron can contribute to oxidative stress through its role in oxygen free radical generation, peroxide reduction, or interactions with superoxide anions. Polyphenols can bind iron primarily because they include galloyl or catechol moieties. In addition, the iron-chelating properties of polyphenols are believed to enhance anticancer effects by facilitating the production of iron-activated redox complexes, thus inducing iron depletion in tumor cells [35]. By directly slowing down the Fenton reaction, chlorination or transition metals like Fe^2+^ can prevent oxidation caused by hydroxyl radicals. Polyphenols do not function in isolation. Polyphenols have been identified as co-antioxidants and play a role in the recovery of vital vitamins [36,38].

In particular, polyphenols have the capacity to enhance the expression of antioxidant enzymes, such as glutathione peroxidase, catalase, and superoxide dismutase, which act on hydroxyperoxides, hydrogen peroxide, and superoxide anions, while concurrently suppressing the activity of pro-oxidant enzymes, including cyclooxygenases and lipoxygenases. Polyphenols may decrease the catalytic function of an enzyme associated with ROS production [37]. They can impede myeloperoxidase’s capacity to oxidize low-density lipoproteins, indicating a possible antiatherosclerotic effect. Their ability to inhibit cyclooxygenases (prostaglandin-endoperoxide synthase), lipoxygenases, and NADPH oxidases is another potential antioxidant advantage [39,40].

Both the direct and indirect oxidant effects of polyphenols are capable of contributing significantly to the reduction in oxidative stress through the aforementioned methods; however, their precise functions at the cellular level can be more complex. A new theory proposes that polyphenols and other phytochemicals may modulate cellular activities via interactions with lipid kinase and protein kinase signaling systems rather than acting as conventional antioxidants that donate electrons or hydrogen. By regulating particular cell signaling pathways, polyphenols in tea may protect cells from oxidative stress. The results suggest that polyphenols in tea may decrease the activities of nuclear factor κB (NF-κB) and IκB kinase (IKK), with EGCG showing the most significant inhibitory effect on IKK, thus enhancing the antioxidant capacity of the body [37]. Polyphenol molecules undergo a transformation into free radicals after contributing an electron or hydrogen atom; thus, at sufficient levels, they may have pro-oxidant effects. Nonetheless, the possibility of pro-oxidant activity occurring in vivo and causing harm to humans is still ambiguous, requiring further research [36]. The pro-oxidative action of polyphenols is contingent upon certain parameters, including their solubility properties, chelating capabilities, metal reducing potential, and pH level at the site of action [41]. The pro-oxidant and pro-apoptotic activities in different tumor cell types may be correlated [42]. Polyphenols function as antioxidants in small amounts; however, in elevated quantities, they exhibit a pro-oxidant impact. This depends on numerous circumstances. A critical aspect is the “seasonal type”, wherein climatic conditions during the growing season of plants, including elevated air temperatures and UV radiation levels, result in increased polyphenol concentrations in plants. Consequently, the content of polyphenols is elevated in animal feed generated during the plant growth season, potentially exerting a pro-oxidant effect. Polyphenols may exert a pro-oxidative impact on other cells under conditions of elevated partial pressure and oxygen concentration [43].

### 3.1. Antioxidant Properties in AML

**Figure 1:**
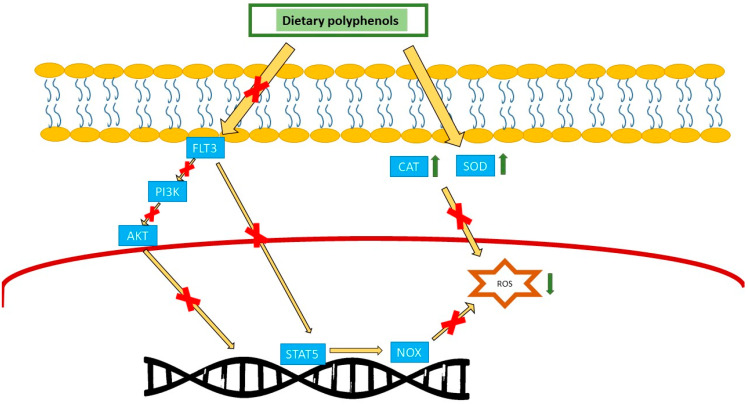
Polyphenols exert their anti-oxidative effect by increasing catalase and superoxide dismutase enzyme and also suppressing the NADPH oxidase pathway, both of which reduce ROS. CAT: catalase, SOD: superoxide dismutase, NOX: NADPH oxidase, ROS: reactive oxygen species, FLT3: FMS-like tyrosine kinase 3, PI3K: phosphoinositide 3-kinase, AKT: AK strain transforming.

Increasing evidence suggests that oxidative stress caused by reactive oxygen species plays a role in the development of leukemia as part of a multistage process, with notable findings described [44]. Numerous mutations occur in hematopoietic stem cells, resulting in genetic instability that enhances the expression of certain protein kinases and transduction proteins, therefore increasing ROS production, which correlates with increased DNA damage. Currently, the use of antioxidant pharmaceuticals is increasingly recognized globally due to their safety and efficacy in mitigating the detrimental effects of free radicals and treating various diseases, including cancer [45]. FMS-like tyrosine kinase-3 internal tandem duplication (FLT3-ITD) represents the primary genetic change in AML. Evidence suggests that the FLT3-ITD-mediated activation of oxidative stress pathways may significantly contribute to drug resistance. We see the downstream FLT3-ITD pathways as important oxidative stress signaling pathways, which include STAT5, PI3K/AKT, and RAS/mitogen activated protein kinase (RAS/MAPK). These subsequent paths can reduce cell death and boost cell growth and survival through regulating genes associated with cell apoptosis and facilitating ROS production through NADPH oxidase (NOX) or other processes (Figure 1). Optimal amounts of ROS can promote cellular proliferation, while elevated levels could induce oxidative damage to DNA and improve genomic instability [46]. Interactions between thioredoxin (TRX) and the c-Jun stimulation domain-binding protein-1 (JAB1) mediated by reactive oxygen species (ROS) are pivotal in the pathobiology and recurrence of AML subtype M5 (AML-M5) [44]. Findings suggest that polyphenols in red grape juice reduce superoxide anion production, providing an alternative rationale for how grape derivatives may alleviate oxidative stress linked to the overproduction of superoxide by nicotinamide adenine dinucleotide phosphate (NADPH) oxidase. This effect is mediated by a reduction in the expression of p47phox, p22phox, and gp91phox at both the mRNA and protein levels, while p67phox expression remains unchanged; all of these are subunits of NADPH oxidase [47]. The consumption of red grape juice (5 μmol/L), a source of dietary polyphenols, may mitigate oxidative stress in AML by influencing NADPH oxidase activity [47]. Green tea (600–900 mg of catechins) polyphenols are suggested to be possible anticancer agents according to their antioxidant characteristics and, recently, their pro-oxidant actions [48]. Tea polyphenols demonstrate significant chelating properties for metal ions, therefore inhibiting the formation of reactive oxygen species [49]. Conversely, the pro-oxidant effects of green tea polyphenols have recently been significantly validated [50]. EGCG, a prominent catechin found in green tea, is a powerful antioxidant linked to the therapeutic benefits of tea [51]. Green tea catechins have the ability to cause apoptosis through intrinsic pathways by modifying the redox system and oxidative stress, as shown by their effect on UF-1 NB4 fresh cells from AML patients. An increase in ROS levels caused by catechins leads to the dissipation of the mitochondrial transmembrane potential, causing the mitochondria to release cytochrome C into the cytoplasm and activate caspase enzymes. Moreover, catechins enhance BAX expression while decreasing BCL-2 and survivin [52].

The harmful effects of EGCG are linked to the autoxidation of the compound, as evidenced by research. This process ultimately leads to the production of H_2_O_2_ and the creation of EGCG auto-oxidation products (EAOPs). Oxygen chemically acquires electrons from EGCG, leading to the creation of superoxide anions, which are subsequently reduced, resulting in the buildup of hydrogen peroxide that inhibits thioredoxin reductase, a potential objective for cancer prophylaxis [50]. In a living organism investigative framework for acute promyelocytic leukemia (APL), notably the PML/RARα fusion (retinoic acid receptor α) model, the consumption of green tea demonstrated the ability to increase reactive oxygen species (ROS) levels in Gr1+ cells in bone marrow and decrease the number of CD34+ and CD117+ cells at the same time. According to these results, green tea can inhibit the proliferation of leukemia cells in living organisms. Reducing the number of cancerous clones that divide within cells is one possible mechanism by which this effect can take place [53]. Another paper indicated that catechins in tea at a dose of 100 μM can target PML–RARα fusion and trigger apoptosis in a murine model via mitochondrial damage and caspase activation [54].

### 3.2. Antioxidant Properties in ALL

A large body of research suggests that reactive oxygen species (ROS) contribute to ALL leukemogenesis. The constitutive activation of many oncogenic kinases is induced by somatic diversity and chromosomal translocations in ALL; these kinases are known to contribute to increased ROS generation in leukemia [55]. Increased lipid peroxidation levels in recently diagnosed ALL patients suggest that this oxidative damage is linked to pathogenesis rather than chemotherapy, which is highly detrimental to the body. Nevertheless, the study of lipid peroxidation throughout childhood is insufficient. Lipid peroxidation products are commonly utilized as biomarkers for oxidative damage; however, identifying oxidant agents is difficult due to their volatility [56]. Membrane lipids are vulnerable to peroxidation. The hydroxyl radical (•OH) serves as a vital reactive species and the initiator of the ROS chain reaction in the process of polyunsaturated lipoperoxidation. Multiple chemicals are produced from the peroxidation of lipid polyunsaturated fatty acids (PUFAs), such as isoprostanes, malondialdehyde (MDA), and 4-hydroxy-2-nonenal (4-HNE) [57]. These molecules serve as indicators in assays for lipid peroxidation [58]. The absorption and accumulation of MDA from dietary sources in human plasma, along with the previously unrecognized effect of red wine polyphenols in inhibiting the assimilation of lipotoxic MDA in humans, lead to a fast buildup of MDA in plasma, reaching a peak concentration 3 h after consumption. MDA is a prevalent cytotoxic substance resulting from lipid peroxidation, predominantly found in foods, particularly meat, and produced endogenously in vivo [59]. The most potent enzyme antioxidants comprise superoxide dismutase (SOD) and catalase (CAT). SOD is an antioxidant enzyme that helps to protect cells from the harm caused by free radicals via the dismutation of superoxide (O_2_ U−) into molecular oxygen (O_2_) and the less reactive hydrogen peroxide (H_2_O_2_). Furthermore, the CAT enzyme effectively facilitates the transformation of H_2_O_2_ into water and molecular oxygen. SOD and CAT activities were shown to be decreased in all cases. CAT activity was reduced in newly diagnosed patients and those in both therapy cohorts. SOD activity was decreased in newly diagnosed patients and those undergoing the induction of remission. This finding signifies a disruption in the protective function of these enzymes against free radicals in ALL [60]. Dietary polyphenols are significant xenobiotics that have physiological implications for human health. The consumption or addition of dietary polyphenols has shown the capacity to re-establish redox equilibrium and mitigate systemic or localized inflammation by enhancing the activity of the antioxidant enzymes SOD and CAT (Figure 1) [58]. Certain phytochemicals may serve as possible chemopreventive drugs against cancer, mostly owing to their ability to stop cancer cells from proliferating. Inconsistent results regarding the antiproliferative effects of dietary phenolic compounds on cancer cells, associated with the production of phenolic-induced H_2_O_2_ in the medium, have been reported [61]. A range of dietary polyphenols, such as gallic acid, ellagic acid, quercetin, myricetin, rutin, kaempferol, resveratrol, catechins, and EGCG, demonstrate double functionalities as both antioxidants and pro-oxidants. The anticancer, anti-obesity, and antibacterial properties of green tea polyphenols (EGCG, (−)-epicatechin-3-gallate (ECG)) are chiefly ascribed to their antioxidant properties, while their detrimental toxic effects result from their pro-oxidative properties [62]. The pro-oxidant effects of EGCG, a key ingredient in tea, become apparent at much higher levels than that required for it to be effective. The pro-oxidant activity of tea polyphenols directly produces ROS and indirectly induces apoptosis and the demise of cancer cells [63]. Apple extract (100 to 500 µM) demonstrates significant pro-oxidant activity in vivo depending on the dosage, treatment duration, plus other nutritional elements. Polyphenols, functioning as pro-oxidant agents, may induce cytotoxic effects on cancerous cells via the production of increased levels of ROS. Elevated levels of ROS ultimately lead to DNA damage when exposed to metal ions, such as copper, resulting in cell death [64]. This pro-oxidant impact could possibly be associated with a pro-apoptotic function in many tumor cell types [42]. The impact of dietary phenolic phytochemicals on carcinogenesis depends on the structural composition of the specific compounds and their dosage. The most common phenolic components in apples are quercetin, epicatechin, procyanidin, chlorogenic acid, and phloretin, excluding gallic acid and EGCG [65,66]. Compared to gallic acid and EGCG, quercetin has a significantly lower impact on ROS generation. In comparison to gallic acid and EGCG, quercetin exhibited a protective effect against H_2_O_2_-induced DNA damage in an in vitro cellular experiment [67]. In addition, the antioxidants quercetin, epicatechin, and vitamin C—which are abundant in apples—have demonstrated protective effects against the inhibition of GJIC by H_2_O_2_. Under the influence of TPA, quercetin and phloretin significantly reduced the production of cyclooxygenase-2 (COX-2) in epidermal cells and murine skin. Apple extracts inhibit tumor promoter-induced carcinogenesis and associated cellular activation in vivo. According to studies of epidemiology, eating more fruits may lower the risk of cancer. It is possible that apples’ antitumor-promoting characteristics—which include reducing inflammation and modulating gap junction intercellular communication at non-cytotoxic doses—are responsible for their cancer chemopreventive actions [61].

NF-κB groups demonstrate persistent activation in ALL, independent of subtype. NF-κB proteins constitute a group of transcription elements that modulate immunological reactions to infections, inflammation, and cell growth, proliferation, and expansion. NF-κB transcription elements regulate the expression of ROS-generating NADPH oxidase enzymes and may additionally increase antioxidant production [55]. Not only does NF-κB activity increase NADPH oxidase expression, thereby augmenting ROS production, but ROS also modulates NF-κB’s transcriptional activity by degrading the inhibitory protein IκB, which plays a crucial role in helping to promote the nuclear transfers and the expression of B genes, thereby establishing a dual forward feedback mechanism and a positive feedback loop [55]. Hydroxycinnamic acids constitute a significant category of polyphenols present in almost all plants. Caffeic acid represents the main example of hydroxycinnamic acids, predominantly occurring within foods as an ester via quinic acid, referred to as chlorogenic acid (5-caffeoylquinic acid). Coffee serves as a notable contributor of chlorogenic acid within human diets. Bioavailability statistics indicate that the biological consequences of chlorogenic acid become evident following its transformation into caffeic acid, highlighting the necessity for further investigations into the effects of this compound. Chlorogenic acid and caffeic acid function as in vitro antioxidants, potentially obstructing the formation of mutant and cancerous N-nitroso compounds by inhibiting the N-nitrosation reaction in in vivo conditions. Moreover, chlorogenic acid has the potential to inhibit DNA damage in vitro by inhibiting the formation of DNA adducts induced by lipid peroxidation and through the inhibition of NF-κB activation, activator protein-1 (AP-1), and mitogen-activated kinases that are influenced by ROS through the enhancement of antioxidant enzyme activity. These experiments indicated that coffee polyphenols are effective chemopreventive agents [68]. However, the maternal consumption of coffee and tea throughout pregnancy is linked to a risk of ALL. Evidence has been observed indicating a heightened risk of pediatric ALL correlated with increased maternal coffee consumption during pregnancy. A similar set of outcomes was observed in instances with a confirmed ETV6-RUNX1 translocation as well as those lacking this translocation; however, the effect estimates have been defined by imprecision owing to the restricted number of cases [69]. Research examining the relationship between coffee and tea consumption and the risk of acute lymphoblastic leukemia (ALL) suggests that slow acetylators may cause an increased risk of ALL in mothers who consume more than two cups of coffee per day. The results concerning the maternal consumption of tea and the likelihood of ALL in children with a sluggish acetylator genotype were similar [70].

## 4. Anticancer Properties of Polyphenols

There is a significant functional connection between diet and cancer. A poor diet can influence the occurrence of specific cancer types by 10% up to 70% (one in five). Numerous epidemiological studies have demonstrated that a daily intake of vegetables and fruits can decrease the occurrence of various kinds of cancer [71]. Numerous epidemiology studies have suggested that a diet enriched in fruits and vegetables, which are rich in polyphenols, is associated with a lower chance of cancer [72]. Research has shown that those who consume a lot of fruits and vegetables have a lower risk of death from cancer. A current meta-analysis of observational research found that rigorous adherence to Mediterranean eating habits, abundant in vegetables and fruits, cut the overall risk of cancer fatality by about 10% [73]. Epidemiology and animal investigations have indicated the significant advantages of polyphenols, indicating preventive effects against many diseases, including malignancies such as leukemia [16]. The anticancer effects of polyphenols include the inhibition of the cell cycle, the stimulation of apoptosis and angiogenesis modification pathways, and the prevention of metastasis. Research indicates that the intake of polyphenol-rich foods may reduce cancer mortality by 10 to 70%. A significant benefit of utilizing polyphenols as anticancer drugs is their low toxicity, safety for consumption, and high accessibility. This may provide opportunities for innovation in medication discovery and could contribute significantly to cancer prevention [71,74]. Fruits abundant in polyphenols, particularly nutritious berries including cranberry, blueberry, blackcurrant, and bilberry, indicate significant chemopreventive and chemotherapeutic consequences against various cancer cell types. Various kinds of polyphenols, encompassing catechins from green tea, have demonstrated anticancer effects on both cancer cell lines and many animal models for carcinogenesis, encompassing chemically produced tumors and transplanted cancer cells [72].

### 4.1. Anticancer Properties in AML

**Figure 2:**
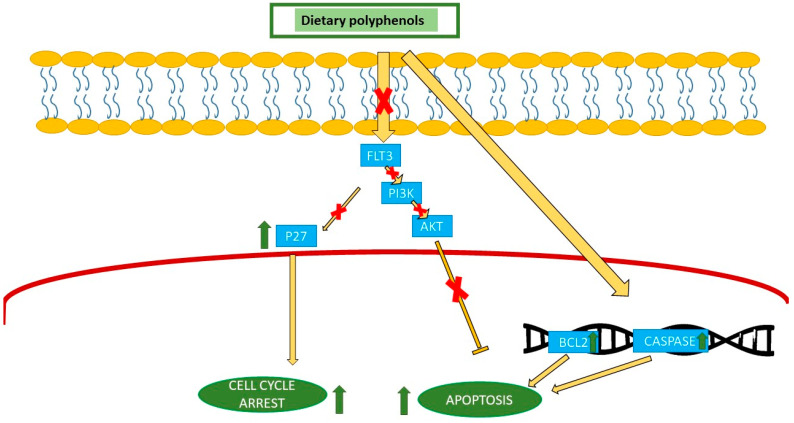
Polyphenols may exert their effects by blocking the FLT3 pathway, which subsequently inhibits the PI3K/Akt signaling pathway. FLT3 pathway inhibition can induce cell cycle arrest by increasing P27 levels. Furthermore, they can increase the expression of pro-apoptotic genes and hence induce apoptosis. FLT3: FMS-like tyrosine kinase 3, PI3K: phosphoinositide 3-kinase, AKT: AK strain transforming.

A significant rate of Tet methylcytosine dioxygenase 2 (TET2) genetic changes is observed in AML, which significantly correlate with a decrease in 5-hydroxymethylcytosine (5hmC) levels, and these mutations are recognized as potential diagnostic and prognostic biomarkers for hematological malignancies. TET2 expression significantly increases in the hematopoietic stem, and it is widely regarded as a gene with tumor-suppressive capabilities. TET2 deletion induces hematologic cancers in mice [75]. TET2 mutations co-occur alongside other mutations in AML, such as TP53 mutations. Ascorbate may provide an advantageous antiproliferative impact on AML cells with TET2 along with TP53 mutations. Vitamin C supports and enhances TET activity, presumably by converting Fe^3+^ to Fe^2+^ within the catalytic cycle. Consequently, ascorbate may function as an epigenetic therapy by enhancing TET2 activity [76]. Juices abundant in vitamin C are also excellent sources of polyphenols. Vitamin C and polyphenols function synergistically [77]. This indicates that the consumption of polyphenols with vitamin C could improve their effectiveness in TET2 activity. NF-κB was identified to be continuously activated in AML [78]. The NF-κB active form engages and collaborates alongside other transcription elements, including STAT3, to regulate the expression of genes [79]. Increased STAT 3 activity makes leukemia cells, including AML, resistant to tyrosine kinase inhibitors (TKIs) [80]. NF-κB facilitates this increase in drug resistance by regulating the expression of the multidrug resistance gene 1 (mdr1) [81]. In AML, the NF-κB cascade is triggered via overexpression or FLT3 functional mutation [82]. FLT3 is a frequently altered gene observed in patients with AML. Clinical investigations indicate that the existence of an FLT3-ITD mutation is significantly correlated with an increased risk of recurrence and diminished overall survival. Consequently, FLT3 activation represents a viable molecular target for acute myeloid leukemia treatment. Green tea polyphenols, such as epigallocatechin (EGC), EGCG, and ECG, inhibit AML cells’ proliferation [83]. Green tea polyphenols reduce proliferation and inhibit FLT3 expression in cells with FLT3 mutations, ultimately leading to the inhibition of subsequent pathways, including PI3K/MAPK and STAT5. The inhibition of FLT3 resulted from the breakdown of the relationship between FLT3-ITD and HSP90, causing a breakdown of FLT3-ITD. These flavonoids synergistically enhanced apoptosis in cells with an FLT3 mutation when administered alongside midostaurin [83]. EGCG, a catechin derived from green tea (Camellia sinensis), has demonstrated several anticancer activities, with a capacity to suppress the growth of APL cells and initiate apoptosis. Moreover, there is an increase in the expression of genes linked to arrests in the cell cycle and differentiation (p27, C/EBPα, PCAF, and C/EBPɛ) (Figure 2). Furthermore, EGCG downregulated the epigenetic regulators DNMT1, HDAC1, and HDAC2, which led to anticancer epigenetic alterations [83]. Treatment with pomegranate induced a notable arrest in S phase in leukemic cell lines, except for human promyelocytic cell line HL-60, where a small percentage of cells were halted in the G0/G1 phase [84]. An investigation into the impact of phenol extract (PE) from virgin olive oil on HL60 cell apoptosis, differentiation, cell cycle arrest, and proliferation revealed that PE hindered HL60 cell proliferation in a manner that was both time- and concentration-dependent. At a concentration of 13.5 mg/L, cell growth was entirely halted [85]. A lack of an effect on topoisomerase II activity and cell proliferation in cancer cell lines was seen with flavanols produced from cocoa. In healthy amounts, these substances should only show limited leukemogenic potential in cases of AML in infants [86]. On myeloid leukemia cell lines, blueberry extracts (25 mg/week) show anti-AML actions, which are mediated via the regulation of protein kinase B (Akt) and extracellular signal regulated kinase (Erk) within the leukemic stem cell fraction [87]. Polyphenol-rich grape pomace and seed extracts have been shown in a range of new research to increase apoptosis and inhibit the growth of leukemia cells [88].

Patients with advanced, progressing malignancies showed treatment efficacy and safety in curcumin clinical trials [89]. Curcumin has been found to have anti-AML effects by rendering AKT inactive; these findings suggest that curcumin could be useful in the treatment of AML [90]. A candidate for future clinical trials could be the herb turmeric. Real-world data from research suggest that all AML patients, despite their age or other risk factors, may have a better chance of a longer survival if they receive Traditional Chinese Medicine (TCM) treatments that include polyphenols in addition to standard therapy [89,91]. For humans, a preliminary clinical trial suggested an intravenous infusion of 1400 mg/m^2^ (2.5 g of quercetin) every week for three weeks at intervals of one week for participants weighing 70 kg [92]. Strong evidence suggests that quercetin, found in tea and coffee, causes AML cells to die by reducing the activity of the VEFG/Akt signaling pathways and triggering mitochondria-mediated apoptosis [93]. Therefore, it may serve as a recommendation for clinical research investigating the anticancer effects of quecerin in AML. Another 13-year follow-up study of 52,000 adults found that green tea protected participants from developing hematologic neoplasms like AML and ALL, particularly AML [94]. Combotastatin, a stilbene derived from the African Bushwillow Combretum caffrum, was combined with cytarabine in a recent phase I trial for relapsed/refractory AML. The results revealed a 19% overall response rate and a considerably longer overall survival for those who obtained complete remission [95].

### 4.2. Anticancer Properties in ALL

Research has shown that certain polyphenols can regulate oxidative stress and lead to apoptosis in cancer cells, therefore halting the disease’s progression [88]. Over 60–85% of newborns diagnosed with ALL have (4;11) (q21;q23) translocation. This translocation is significantly linked to a chemotherapy-resistant phenotype and an adverse survival prognosis. Consequently, this variant of leukemia has been classified into the high-risk leukemia subgroup. The persistence of this type of leukemia despite standard chemotherapy treatments highlights the need for constant research into potential new preventative and therapeutic measures. Cell lines generated from individuals who had t (4;11) revealed that strawberry substances induced cell death by modifying the potential of the mitochondrial membrane and caspase-3 activation. Berry fruits such as blackberries, green, red, blue, and black grapes, and raspberries are rich in phytochemicals that warrant investigation because of their possible effectiveness in avoiding or treating this particular form of leukemia [96]. Several studies are being conducted to develop viable treatments for leukemia. Extensive research carried out in recent decades has shown that grape pomace and seed extracts, rich in polyphenols, induce apoptosis and inhibit the development of leukemic cells [88].

Green tea catechins are bioactive polyphenolic chemicals that have attracted significant interest owing to their numerous biological functions and possible health benefits. EGCG has been identified as a powerful apoptosis inducer, functioning via processes including the activation of caspase, the modulation of Bcl-2-related proteins, the impairment of survival signals, and the adjustment of redox equilibrium, thereby inducing oxidative stress. In addition, recent evidence indicates that catechins in green tea may impact epigenetic alterations, including histone modifications and DNA methylation. In addition to their apoptotic effects, signaling via ROS influences, and the reverse process of epigenetic alterations, green tea catechins have demonstrated promising outcomes in facilitating leukemia cell development. All catechins (or polyphenols) in green tea induce apoptosis in various kinds of cancer, both in vitro and in vivo, including lymphoid leukemia [88]. It was observed that when four patients diagnosed with low-grade cancers of B cells independently began the oral consumption of items comprising tea polyphenols, they showed a measurable clinical response [97]. These investigations indicate that green tea catechin derivatives are secure and well tolerated at the examined dosages in both healthy individuals and leukemic patients while also being cost-effective and efficient (Figure 2) [88].

A study by Kitsati et al. clarified the capacity of olive oil components, especially hydroxytyrosol, to regulate intracellular iron homeostasis and subsequently affect redox-mediated signal transduction. Cells belonging to the human T-lymphocytic cell line JURKAT were utilized in this research. In addition, DNA damage was classified into five groups using single-cell gel electrophoresis (comet assay) [98]. According to Parra-Perez’s research, JURKAT cells undergo cell cycle arrest after being administered with hydroxytyrosol. In comparison to the control group, they discovered a marked decrease in phase S cells and an enormous uptick in G0/G1-phase cells. ROS production and cell death are both accelerated by hydroxytyrosol. Lowering the expression levels of KSR1 and C-Myc led to a marked reduction in cell differentiation and an increase in cell death in JURKAT cells, respectively [98,99].

A clinical experiment was carried out with 24 children, ranging in age from 4 to 6, who were diagnosed with acute lymphoblastic leukemia. Olive oil has been found to be effective in preventing chemotherapy-induced oral mucositis (OM) in children with ALL [100]. Thirty children who tested positive for ALL were monitored for a year. For one month, participants took 500 mg of curcumin orally twice a day in capsule form. The results from this preliminary study suggest that curcumin may be useful as a dietary supplement for children with ALL [101].

## 5. Anti-Inflammatory Properties of Polyphenols

**Figure 3:**
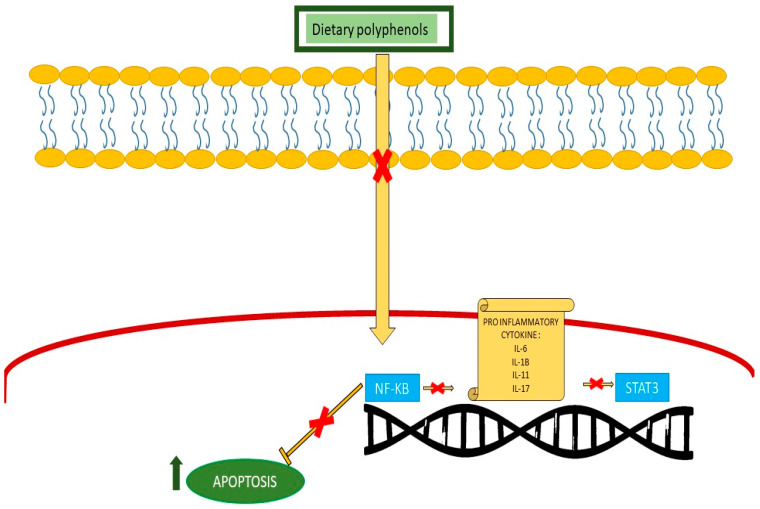
Dietary polyphenols can inhibit the NF-κB pathway, which is activated in inflammatory conditions, thereby suppressing the generation of inflammatory cytokines and inhibiting STAT3. The NF-κB pathway suppresses apoptosis; hence, the polyphenol method induces apoptosis in leukemic cells. NF-κB: nuclear factor kappa B.

Polyphenols trigger inflammatory responses through many pathways, such as modulating cytokine synthesis, impacting immune cell populations, the regulation of pro-inflammatory gene expression, and their antioxidant capacities [39,102,103]. Polyphenol activity is primarily located in the stomach, where it initiates immunoprotective and anti-inflammatory actions, hence providing systemic anti-inflammatory effects [104,105]. Oxidative stress-induced inflammation is exacerbated by the activation of NF-κB and AP-1. It influences numerous cellular signaling pathways, resulting in the production of inflammatory mediators and chromatin modification. The latter facilitates the expression of pro-inflammatory genes, including interleukin-1 beta (IL-1β), IL-8, tumor necrosis factor alpha (TNF-α), and inducible nitric oxide synthase (iNOS) [30]. The excessive generation of mitochondrial reactive oxygen species (ROS) stimulates the manufacturing of pro-inflammatory cytokines with the activation of the NLRP3 inflammasome, which is a part of the nucleotide-binding oligomerization domain, the leucine-rich repeat-containing gene family, and pyrin domain-containing proteins [106]. NLRP3 is a pivotal component that interconnects the signaling pathways of redox response and inflammation. The NLRP3 inflammasome is stimulated by cytoplasmic ROS, leading to an enhanced release of inflammatory mediators. The activation of Toll-like receptor (TLR)-1-mediated inflammatory signaling occurs when the inflammasome triggers the release of the cytokine IL-1β from the cytoplasm into the extracellular environment. Although TLRs are most commonly associated with the immune system, they have been found in various other organs, such as the gastrointestinal tract and liver. The production of cytokines such as interleukin (IL)-1β, IL-6, IL-2, tumor necrosis factor (TNF)-α, and interferon (IFN)-γ is triggered when IL-1 activates Toll-like receptor-1, which in turn triggers pro-inflammatory signaling transductions mediated by NF-κB and MAPK. This series of events ultimately leads to the enhancement of inflammatory processes, leading to systemic inflammation. Multiple in vitro and in vivo investigations have elucidated which dietary phenolic compounds confer protective benefits against inflammation through the modulation of NLRP3 activation [58]. The adverse consequences of oxidative stress can be mitigated by the anti-inflammatory or antioxidant properties of dietary polyphenols, comprising resveratrol and curcumin, both in vivo and in vitro. For instance, resveratrol suppressed pro-inflammatory gene expression by inhibiting inhibitory κB (IκB), hence obstructing NF-κB transactivation and reinstating trans-repressive processes with the stimulation of histone deacetylases, which are found in RAW 264.7 cells [30]. In mouse and rat macrophages, polyphenols suppress COX, deactivate peroxisome proliferator-activated receptor gamma (PPARγ), and stimulate endothelial nitric oxide synthase (eNOS) [107]. In addition, they reduce the levels of inflammatory mediators like prostaglandins and leukotrienes, as well as inflammatory cytokines like TNF and IL-1 and adhesion molecules like ICAM-1 and VCAM-1 in human umbilical vein tissues. Furthermore, they block the action of specific inflammatory enzymes, including COX in mice, lipoxygenase (LOX) in human endothelial cells, mitogen-activated protein kinase (MAPK), and the inhibitor of kappa kinase (IKK). In addition, curcumin lowers the activity of NF-κB and STAT3 while simultaneously lowering the expression of TLR-2 and TLR-4. Its effect on adult male rats involves increasing the expression of PPARγ (peroxisome proliferator-activated receptor gamma) (Figure 3) [104]. It suppresses the production of several chemokines and cytokines that promote inflammation, including TNFα, IL-6, IL-1β, IL-8, and MCP-1 (monocyte chemoattractant protein 1), in several kinds of cells, including activated primary macrophages with lipopolysaccharide from mice, activated human mast cell lines, activated human astrocytes, human synovial cells, and human peripheral blood mononuclear cells [108]. In its immunomodulatory role, polyphenol utilization is linked to a direct alteration in the quantity and maturation of particular immune cells. The oral consumption of polyphenols derived from fruit correlates with an elevation in natural killer (NK) cells, T helper 1 (Th1) cells, macrophages, and dendritic cells (DCs) within Peyer’s patches of the spleen in male C3H/HeN mice [102]. In humans, polyphenols can increase the number of regulatory T cells, characterized by their profile (Foxp3+, CD4+, CD25+) and implicated in immune tolerance and autoimmunity control [109].

### 5.1. Anti-Inflammatory Properties in AML

The deactivation of Tet2 or DNMT3A, two frequently altered genes among individuals with clonal hematopoiesis in myeloid tumors, is related to an outbreak of an inflammatory condition. Subsequently, through the enhancement of cell cycle efficiency or the facilitation of apoptosis evasion, the stimulation of inflammatory pathways can hasten the progression to AML and promote the proliferation of mutated clones [110]. Turmeric contains the polyphenolic chemical curcumin, which shows great promise as an epigenetic regulator and is well known for its substantial anti-inflammatory effects. By blocking DNA methyl transferases (DNMTs), curcumin is able to regulate epigenetic processes [111]. This indicates that the natural substance curcumin has significant anti-inflammatory effects and promotes apoptosis, suggesting that it could be a potent polyphenol to induce apoptosis through the reduction in inflammation [112]. Inflammation drives the pathogenesis of myeloid malignancies across the spectrum of these diseases, including AML. The overexpression of inflammatory cytokines, including IL-1β, IL-6, and TNF-α, as well as growth factors like insulin-like growth factor (IGF-1), is common in AML. These substances play an essential role in the leukemic stem cell niche by activating NF-κB, which induces ferritin synthesis [10]. Malignant transformation is significantly linked to the dysregulation of NF-κB. Cancer cells become resistant to apoptosis, inflammation is reduced, and cell proliferation is accelerated. This encourages the spread of tumor cells by triggering angiogenesis. Both experimental animal models of AML and people with AML frequently exhibit constitutive NF-κB activation [113]. Furthermore, AML that relapses or develops as a result of Fanconi anemia is linked to inflammatory cytokines [114,115]. Recent evidence linking the development of several types of leukemia to the degree of angiogenesis has established the involvement of angiogenesis in the proliferation and maintenance of leukemic cells [116]. It is widely acknowledged that inflammatory cells play a role in tumor angiogenesis through the release of matrix metalloproteinase (MMP) and that circulating inflammatory cells such neutrophils, tumor-associated macrophages (TAM), and activated T lymphocytes might contribute to this process. This study’s findings suggest that some catechins found in green tea may have antiangiogenic and chemopreventive properties that could be useful in the fight against macrophage-like leukemia cells, the amelioration of tumor-associated inflammation, and improvements in existing chemotherapeutic interventions [117]. In order to reduce inflammation and apoptotic resistance, olive polyphenols indirectly decrease NF-κB expression through controlling the production of inflammatory cytokines and proteins involved in the apoptosis pathway. Research showing that inhibiting NF-κB expression with the chemical BMS-345541 changed the expression of genes essential for leukemogenesis in AML cells generated from patients clearly established the role of NF-κB in leukemogenesis [118,119]. Among the novel anti-inflammatory drugs with potential benefits for the treatment of AML are those derived from blueberry extracts [120,121]. Blueberry extracts have antibacterial and anti-inflammatory properties. By controlling the levels of interleukins 1β, 6, and -12, which are pro-inflammatory cytokines, through suppressing the expression of their genes, blueberry polyphenols demonstrate anti-inflammatory action. Additionally, they can block neutral sphingomyelinase and NADPH oxidase-mediated pro-inflammatory signaling pathways [122]. According to research, blueberry extracts can lower blood sugar levels by regulating the inflammatory pathway and increasing the expression of GLUT-2 and PPARγ through the interaction of caffeoylquinic acid derivatives and quercetin glycosides [123]. Lowbush blueberries contain 0.44 mg/g of chlorogenic acid, and the combination of phenolic acids in these fruits has anti-inflammatory effects by regulating NF-κB activation and, at high doses, creating inflammatory cytokines (Figure 3) [124].

The safety and potential improvement in the immune system in elderly AML-MRC patients by reducing lymphocytosis and the absolute number of circulating Treg cells, as well as IL-10 and TGF-β serum levels, which are associated with inflammation, were demonstrated in a clinical study in which patients with myelodysplasia-related changes were given oral doses of GT extract (1000 mg/day) either alone or in combination with low-dose cytarabine chemotherapy [125].

### 5.2. Anti-Inflammatory Properties in ALL

Inflammation is commonly linked to the initiation and advancement of numerous malignancies by activating cellular signals which induce DNA damage along with other epigenetic modifications [126]. This is followed by an escalation in the infiltration of inflammatory cells and the stimulation of the immune cell signaling molecules, NF-κB and STATs [127]. The suppression of eicosanoid-generating enzymes—LOX and cyclooxygenase (COX)—is the key mechanism by which polyphenols, especially flavonoids and anthocyanins, exert their anti-inflammatory effects. Several illnesses may be affected by the enzyme conversion of polyunsaturated fatty acids, especially arachidonic acid [128].

In ALL, the majority of patients exhibit constitutive initiation of the traditional NF-κB pathway via RelA/p50 complexes, which are essential for enhancing the lifespan of ALL cells by preventing apoptosis or encouraging the proliferation of cells [78,129]. It has been found that the majority of cases involve kinases that phosphorylate IĸBα [129]. The triggering of NF-κB-dependent transcription is enhanced in ALL and chronic myeloid leukemia (CML) due to the production of Bcr-Abl and the improvement in the conversion function of the RelA/p65 monomer of NF-κB [16].

Consistent use of olive oil lowers inflammation, according to a new meta-analysis of RCTs. Specifically, C-reactive protein, tumor necrosis factor, and interleukin-6 were all found to be reduced in plasma during these trials [98,105].

## 6. Microencapsulated and Nano-Encapsulated Forms of Dietary Polyphenols’ Impact on AML and ALL

The consumption of polyphenols initiates a number of events in the digestive system that may diminish their medicinal efficacy. The role of polyphenols in the body may be constrained by their low hydrophilicity, weak intrinsic solubility, or physical and chemical instability. Moreover, these physicochemical features may hinder the bioavailability of polyphenols, resulting in diminished absorption, restricted distribution within the body, insufficient penetration, and buildup in human organs. For polyphenols to exert efficacy, their properties and chemical structure must be maintained throughout the digestive process, their intestinal absorption, their entry into the bloodstream, and their traversal of the plasma or intracellular membranes to facilitate their biological activity. They must, of course, reach the target organs in the correct medication dosage to fulfill their primary purpose [130,131]. Consequently, strategies exist to maintain the integrity of polyphenols and, as a result, their efficacy within the body. Currently, nanoencapsulation is utilized for the delivery of bioactive compounds, including polyphenols. This technique safeguards these chemicals against hydrolytic enzymes, oxidation, and other severe physical and chemical conditions throughout the gastrointestinal and systemic pathways while also addressing the challenges regarding the accessibility and bioavailability of polyphenols. In this context, microencapsulation is utilized to preserve phenolic chemicals by encapsulating them in a matrix that facilitates their targeted release during gastrointestinal digestion [132,133].

Nanoencapsulation is a technique that uses submicroscopic technology to encase active substances, whether they are solid or liquid, in a colloidal system that is 10 to 1000 nanometers in size, known as a nanoparticle (NP). The distribution of its active components determines whether a nanoparticle is a nanosphere or a nanocapsule. A nanosphere is a type of nanoparticle in which the active ingredients are either linked to polymer chains or evenly deposited onto the nanoparticle’s surface. Nanocapsules are obtained when these materials are placed in a core or cavity and then surrounded with a polymeric membrane. One or more polymers, either natural or synthetic, can be used to make either kind of nanoparticle [134]. Particles of sizes varying from a few nanometers to several millimeters can be created by enclosing a substance in a coating in the mechanical and physicochemical process known as microencapsulation. Core materials are contained within encapsulated compounds, which are dispersed in a matrix referred to as a coating or shell [133]. The majority of research indicates that polyphenols are more bioavailable and easier to reach when they are microencapsulated [135]. Bioactive chemicals, such as polyphenols, are protected from outside influences through nanoencapsulation, which enhances their stability and prolongs their half-life. In their role as therapeutic agent carriers, nanoparticles improve solubility and chemical stability, allow for the regulated release of active substances after ingestion, and ultimately accomplish the desired outcome [136]. However, nanoparticles can penetrate tissues through the minuscule capillaries and pass through the intestinal epithelial pores because of their subcellular size, which is similar to biomolecules. By targeting particular organs and tissues, this capability enhances the bioavailability of medicinal medicines [137]. Nanoparticles synthesized from natural polymers have not been found to induce cellular toxicity according to toxicological studies [132,135].

Research has been undertaken on the encapsulation of dietary polyphenols. Researchers have investigated the anti-inflammatory properties of cherry extract (*Prunus avium* L.). They analyzed substances abundant in polyphenols, including quercetin and cyanidin-3-glucoside. The microencapsulation of these extracts in fourth-generation chitosan ammonium nanoparticles markedly diminished the concentrations of TNF-α, interleukin-6 (IL-6), nitric oxide (NO), and COX-2-dependent prostaglandin E2 while elevating the level of the anti-inflammatory interleukin-10 (IL-10) [138]. A separate study indicated that after 14 days of administering 160 mg of curcumin-containing nanoparticles, there was a notable decrease in mRNA expression and the release of cytokines IL-1β and IL-6 in COVID-19 patients [139]. A study showed that encapsulating resveratrol with carboxymethyl chitosan (CMC) cross-linked with CaCl2 markedly maintains the antioxidant activity of resveratrol in comparison to free resveratrol [140]. Additional research has been undertaken on the anticancer characteristics of these substances, demonstrating their ability to impede cancer progression by reducing growth, proliferation, metastasis, and angiogenesis [140].

The co-encapsulation of two substances can also be efficacious. Flavonoids, a category of polyphenols, have been identified as possible anticancer agents owing to their antitumor and anticancer characteristics. Nonetheless, their lipophilic characteristics and chemical instability necessitate the use of specialized technologies, such as nanoparticles. The amalgamation of flavonoids with anticancer pharmaceuticals in nanoparticles can provide synergistic benefits and diminish treatment resistance [141]. A separate study showed that the nanoencapsulation of a combination of bioactive chemicals within a single particle can serve as an effective technique for oral antioxidant supplementation, enhancing physicochemical potential and preserving biological qualities. This study revealed the simultaneous loading of quercetin and EGCG into lecithin nanoparticles, showcasing their synergistic antioxidant activity [142].

There is some evidence of effective nanodrugs that deliver leukemia drugs using encapsulation technology. In the case of acute myeloid leukemia (AML), one liposomal formulation that has received FDA approval is Vyxeos. This formulation co-delivers cytarabine and daunorubicin in a fixed molar ratio of 5:1 [143]. Marquibo, a liposomal version of vincristine sulfate, is another nano medicine that has been approved by the FDA for the treatment of recurrent ALL in adults. It was intended to prolong the medication’s circulation in the bloodstream compared to free vincristine. It contains sphingomyelin/cholesterol liposomes with an aqueous inner core and 0.16 mg/mL of vincristine [144]. In the context of polyphenol encapsulation, another study discovered that EGCG nanoparticles suppressed tumor development activity and decreased the growth of APL HL-60 cells [145]. When evaluating their anticarcinogenic properties in human cancer cells such as THP-1 (acute monocytic leukemia), another study found that polymer-based nanoparticles containing EGCG and theaflavin (TF) alone or together with the chemotherapy drug cisplatin were more effective than EGCG/TF alone [146]. Sorafenib and HA-EGCG self-assembled in water to create sorafenib-loaded micellar nanocomplex (Sora-MNC), a nanomedicine for AML treatment that targeted bone marrow. When given in the same amounts, sora-MNC formulations killed FLT3-mutated AML cells a lot more effectively than free sorafenib [147]. Cocoa procyanidins (CPs) extract of the cocoa beans have been embedded in gelatin-chitosan nanoparticles (CSNPs). The findings revealed that the encapsulation of the CPs extract enhanced its stability, exhibiting significant apoptotic impacts for human acute leukemia lines [148].

According to the results obtained, encapsulating dietary polyphenols can enhance the treatment of AML and ALL by mitigating inflammation, diminishing oxidative effects, and inhibiting cell formation, development, and proliferation. Furthermore, the co-encapsulation of polyphenols with standard pharmacological agents for acute leukemia treatment may augment the efficacy of these treatments. This method is very efficacious for individuals who are unresponsive to treatment. Utilizing polyphenols in nanoparticles is advised to augment and enrich food and beverages, as well as in the formulation of dietary supplements for prevention and treatment purposes [132]. This may serve as a methodology for forthcoming investigations into the treatment of acute leukemias.

## 7. Conclusions

Phenolic compounds act as antioxidants by donating electrons or hydrogen atoms to free radicals, transforming them into harmless molecules. ROS are essential to cancer development and progression; their overproduction can induce oxidative stress, resulting in cellular damage and promoting the proliferation and survival of cancer cells. We know about the capacity of ROS to activate numerous signal transduction pathways, such as NF-κB and activator protein-1, which in turn triggers the transcription of genes governing the cellular development pathways. Berry fruits, including strawberries, blueberries, and grapes, as well as green tea are abundant in phenolic compounds that contain antioxidant qualities and antiproliferative effects. The degree of effectiveness of polyphenols as antioxidants depends on how much they are hydroxylated, whether they have sugar moieties, and how their hydroxyl groups react with other molecules. ROS contribute to the onset of AML and ALL, and evidence indicates that the consumption of polyphenols, such as those found in green tea, grapes, and extra virgin olive oil, which are abundant in bioactive substances with antioxidant properties, significantly inhibits ALL cell growth in vitro due to their beneficial attributes, including antioxidant effects, anti-inflammatory properties, and regulation of epigenetic modifications (Table 1). The evaluation of dietary polyphenols reveals intriguing results regarding their influence on this condition, demonstrating both antioxidant and pro-oxidative capabilities, as well as an iron-chelating mechanism (Table 1). Although this pro-oxidant role can be detrimental to healthy bodily functions, it can be helpful in eliminating tumor cells by activating caspase enzymes, upregulating pro-apoptotic genes including Bax, and activating the intrinsic apoptotic pathway (Table 1).

Inflammation is linked to the initiation and advancement of the majority of malignancies, leading to DNA damage and epigenetic alterations. Polyphenols, especially flavonoids and anthocyanins, exhibit anti-inflammatory effects by blocking enzymes that produce eicosanoids, including LOX and COX. The results obtained from previous studies indicate that inflammation is a critical factor in tumor growth and AML relapse. The activation of NF-κB is a crucial signaling mechanism associated with inflammation, resistance to apoptosis, and tumorigenesis; thus, the downregulation of this pathway is of particular importance (Table 1). In addition, inflammatory cytokines participate in angiogenesis and affect the tumor microenvironment. Results indicate that the intake of dietary polyphenols, including green tea, olive oil, and blueberry, can suppress inflammatory pathways. The inhibition of the NF-κB pathway by dietary polyphenols may alter the secretion of pro-inflammatory cytokines, thus reducing angiogenesis and resistance to apoptosis, ultimately leading to a decrease in the progression of AML (Table 1). In ALL, most patients exhibit the continuous involvement of the classical NF-κB pathway, essential for cellular survival. The regular consumption of olive oil reduces inflammation, resulting in a reduction in the levels of three plasma inflammatory mediators. Hydroxytyrosol, a crucial fatty acid, has been demonstrated to inhibit the production of pro-inflammatory cytokines, iNOS, and NF-κB, hence diminishing the formation of thromboxane B2 and prostaglandin E2 (Table 1). This impact may be associated with a reduction in chronic diseases such as cancer.

The results indicate that polyphenols possess anticancer properties. There is evidence of the effects of polyphenols on tumor formation through various mechanisms. The findings illustrate the ability of polyphenols to modulate FLT3 expression, which is suppressed, resulting in apoptosis via altering downstream pathways that include PI3K/MAPK and triggering cell cycle arrest via the overexpression of genes such as p27 (Table 1). In addition to the aforementioned results, dietary polyphenols combined with traditional AML treatment, such as midostaurin, can have beneficial effects, particularly in FLT3 mutant patients, which induce apoptosis (Table 1). Research demonstrates that the intake of polyphenols, particularly from sources like green tea, grapes, and extra virgin olive oil, which are abundant in bioactive compounds with antioxidant characteristics, markedly suppresses the proliferation of ALL cells in vitro. This effect is attributed to their advantageous qualities, encompassing antioxidant effects, anti-inflammatory properties, and the modulation of epigenetic changes (Table 1). Although berry fruits, such as fresh blackberries, blueberries, red, green, and black grapes, and raspberries, are rich in phytochemicals that should be investigated for their potential in preventing or treating ALL, strawberry components caused cellular apoptosis by altering mitochondrial membrane potential and activating caspase-3 (Table 1). Furthermore, while the consumption of polyphenols has favorable effects, some exhibit no impact on tumor cells, underscoring the need for further experimentation.

The challenges associated with polyphenols include their poor absorption, limited solubility, and structural alterations during gastrointestinal transit, prompting the creation of novel delivery strategies, such as encapsulation. These techniques (microencapsulation and nanoencapsulation) maintain the integrity of these substances and shield them from environmental factors, leading to enhanced absorption and a higher quantity reaching the target organ. Furthermore, co-encapsulation of these compounds with other phenolic substances or frequently utilized pharmaceuticals in therapy may produce favorable outcomes.

In conclusion, dietary polyphenols may enhance the treatment of acute leukemia when used with conventional chemotherapy drugs, owing to their pro-apoptotic and pro-oxidant characteristics that augment cancer cell sensitivity to chemotherapy and optimize molecular pathways. They also hold promise for chemoprevention, exhibit antioxidant and anti-inflammatory characteristics, and augment the efficiency of anticancer agents. Moreover, a notable benefit of employing polyphenols as anticancer medicines is their minimal toxicity, safety for consumption, and high accessibility, which can provide opportunities for innovation in drug discovery and contribute significantly to cancer prevention.

Nonetheless, despite the current information about the diverse effects of polyphenols in the treatment of acute leukemias, additional research is required in this domain. To assess the efficacy of dietary polyphenols in treating acute leukemia, it is essential to investigate both their therapeutic effects and potential side effects. Consequently, the impact of various polyphenols on acute leukemias, particularly on mutations, translocations, and other characteristics particular to these malignancies, warrants investigation. Furthermore, it is essential to ascertain the effective dosage of these polyphenols for treatment and to evaluate whether changing amounts may provide different results. For instance, investigating which dosages achieve an oxidative and inflammatory equilibrium is crucial, particularly given the pro-oxidant characteristics of polyphenols and what pharmaceutical dosages can be synergistically mixed with standard therapies to augment their efficacy without inducing side reactions. Occasionally, it has been noted that certain alternative treatments, despite their positive effects on the targeted ailment, have resulted in detrimental consequences on the body and have even precipitated the emergence of additional diseases. They have also adversely impacted other pre-existing disorders in individuals. Consequently, this matter has to be taken into account regarding polyphenol ingestion. It is crucial for polyphenolic chemicals to not only manage the disease but also inhibit the progression of other cancers to acute leukemia. This matter warrants consideration, as not all polyphenols may be efficacious in treatment and require examination.

## Figures and Tables

**Table 1:** An overview of the properties of dietary polyphenols, focusing on their anticancer, anti-inflammatory, and antioxidant effects.

Properties	Polyphenols	Type of Leukemia	Mechanism
Antioxidant	Green tea, olive oil, grapes	ALL	Cell growth inhibition and epigenetic modification.
	Dietary polyphenol	AML/ALL	Iron-chelating, increase in pro-apoptotic genes, and increase in caspase.
Anticancer	Polyphenols combined with chemotherapy treatment	AML/ALL	Increase in chemotherapy sensitivity and modulating molecular pathways.
	Green tea	AML	Cell cycle arrest by P27, FLT3 inhibition, and apoptosis by PI3K/AKT.
	Green tea, grapes, virgin olive oil, strawberry	ALL	Cell growth inhibition, epigenetic modification, and induce apoptosis.
Anti-inflammatory	Green tea, olive, blueberry	ALL/AML	Suppress NF-κB pathway, induce apoptosis, and reduce inflammatory cytokine.
	Olive oil, hydroxytyrosol	ALL	Reducing thromboxane B2 and prostaglandin E2 and pro-inflammatory mediators.

ALL: acute lymphoblastic leukemia; AML: acute myeloid leukemia; FLT3: FMS-like tyrosine kinase 3; PI3K: phosphoinositide 3-kinase; AKT: AK strain transforming; NF-κB: nuclear factor (NF)-κB.

## Data Availability

We used PubMed, SCOPUS, and ScienceDirect databases to screen articles for this review. We did not report any data.
